# Blood leukocyte Alu and LINE-1 methylation and gastric cancer risk in the Shanghai Women's Health Study

**DOI:** 10.1038/bjc.2011.562

**Published:** 2011-12-15

**Authors:** Y Gao, A Baccarelli, X O Shu, B-T Ji, K Yu, L Tarantini, G Yang, H-L Li, L Hou, N Rothman, W Zheng, Y-T Gao, W-H Chow

**Affiliations:** 1 Genetic Epidemiology Branch, Division of Cancer Epidemiology and Genetics, National Cancer Institute, National Institutes of Health, 6120 Executive Boulevard, Building. EPS/Room 7110, NIH/NCI, Bethesda, MD 20892-7236, USA; 2 Department of Environmental Health, Harvard School of Public Health, Boston, MA 02115, USA; 3 Vanderbilt Epidemiology Center, Vanderbilt University School of Medicine, Nashville, TN 37203, USA; 4 Department of Environmental and Occupational Health, Center of Molecular and Genetic Epidemiology, Maggiore Policlinico Hospital, Mangiagalli and Regina Elena IRCCS Foundation, University of Milan, Milan 20122, Italy; 5 Department of Preventive Medicine, Feinberg School of Medicine, Northwestern University, Chicago, IL 60611, USA; 6 The Robert H. Lurie Comprehensive Cancer Center, Feinberg School of Medicine, Northwestern University, Chicago, IL 60611, USA; 7 Department of Epidemiology, Shanghai Cancer Institute, Shanghai 200032, China

**Keywords:** gastric cancer, DNA methylation, leukocyte

## Abstract

**Background:**

Recent data suggest a link between blood leukocyte DNA methylation, and cancer risk. However, reports on DNA methylation from a prospective study are unavailable for gastric cancer.

**Methods:**

We explored the association between methylation in pre-diagnostic blood leukocyte DNA and gastric cancer risk in a case–control study nested in the prospective Shanghai Women's Health Study cohort. Incident gastric cancer cases (*n*=192) and matched controls (*n*=384) were included in the study. Methylation of Alu and long interspersed nucleotide elements (LINE)-1 were evaluated using bisulphite pyrosequencing. Odds ratios (ORs) and 95% confidence intervals (CI) were calculated from logistic regression adjusting for potential confounders.

**Results:**

Alu methylation was inversely associated with gastric cancer risk, mainly among cases diagnosed one or more years after blood collection. After excluding cases diagnosed during the first year of follow-up, the ORs for the third, second, and first quartiles of Alu methylation compared with the highest quartile were 2.43 (1.43–4.13), 1.47(0.85–2.57), and 2.22 (1.28–3.84), respectively. This association appeared to be modified by dietary intake, particularly isoflavone. In contrast, LINE-1 methylation levels were not associated with gastric cancer risk.

**Conclusion:**

Evidence from this prospective study is consistent with the hypothesis that DNA hypomethylation in blood leukocytes may be related to cancer risk, including risk of gastric cancer.

Although its incidence has declined in the past decades, gastric cancer is still the fourth most commonly diagnosed cancer and the second most common cause of cancer death in the world (Jemal *et al*, 2011). *Helicobacter pylori* (*H. pylori*) infection, dietary factors (e.g., low vegetable and fruit intake, and high salt intake), and smoking have been associated with an elevated risk of this disease (Schottenfeld and Fraumeni, 2006). Genetic variations also have been suggested to have a role in gastric cancer development (Gao *et al*, 2009; Peleteiro *et al*, 2010). However, the molecular aetiology of gastric cancer remains unclear and we lack biomarkers for risk prediction for this disease. Epigenetic changes, which may represent an archive of past carcinogenic exposure (Jirtle and Skinner, 2007; Skinner, 2011), could be considered such candidates.

DNA methylation, the covalent addition of a methyl group, is an epigenetic event that affects cell function by altering gene expression. The cytosine nucleotide is the major target of methylation reaction and the product is usually 5-methyl-deoxycytidine (5-mdC). Changes in DNA methylation patterns are hypothesised to be involved in carcinogenesis (Ehrlich, 2002). Global DNA methylation, that is, the degree of methyl attachment to cytosine residues of DNA sequences across the genome, is typically reduced in a wide range of tumours (a phenomenon termed as ‘global hypomethylation’), including gastric cancer (Cravo *et al*, 1996; Hsiung *et al*, 2007; Moore *et al*, 2008; Guerrero-Preston *et al*, 2009). Global DNA methylation was decreased among human gastric cancer cell lines (Kaneda *et al*, 2004; Jiang *et al*, 2008), which was usually associated with chromosome instability (Watanabe and Maekawa, 2010). Significantly decreased global methylation has also been shown in gastric cancer tissue (Chalitchagorn *et al*, 2004; Suzuki *et al*, 2006) and pre-cancer lesions (Cravo *et al*, 1996; Yamamoto *et al*, 2008). Crave *et al* (1996) observed a graduated decrease in global DNA methylation in gastric specimens from normal, superficial gastritis, chronic atrophic gastritis with intestinal metaplasia, to intestinal type of gastric carcinoma.

Repetitive elements represent a large portion of the human genome and contain much of the CpG methylation found in normal human postnatal somatic tissues (Yang *et al*, 2004). Long interspersed nucleotide elements (LINE) make up about 15% of human genome with ∼0.5 million copies, and Alu-repetitive elements make up about 10% of human genome with 1.4 million copies. It is estimated that more than a third of DNA methylation occurs in the repetitive sequences (Weisenberger *et al*, 2005), and methylation levels of LINE-1 and Alu have been used as proxies for global DNA methylation status (Bollati *et al*, 2007, 2009; Tarantini *et al*, 2009).

Many studies on DNA methylation and gastric cancer to date have been conducted by comparing tumour tissue DNA with adjacent normal tissue DNA from cancer patients, and found decreased global or repetitive elements DNA methylation in tumour tissue compared with adjacent normal tissue (Chalitchagorn *et al*, 2004; Suzuki *et al*, 2006). Relatively fewer studies have been conducted on DNA methylation change in other non-target tissues (Chalitchagorn *et al*, 2004; Board *et al*, 2008). Methylation status of some CpG sites could be passed from previous generations as an inherited marker (Kile *et al*, 2010), or affected by aging or environmental exposures as a whole organism throughout life (Christensen *et al*, 2009). Changes of methylation in leukocyte DNA have been shown to parallel other somatic tissues (McKay *et al*, 2011), and have been linked to susceptibility of certain cancers (Marsit *et al*, 2011). Certainly, we could not rule out the possibility that different tissues have different response to environmental exposure and that methylation status in leukocytes may not fully reflect the changes in the target tissue (McKay *et al*, 2011). We previously observed suggestive associations between hypomethylation in Alu and LINE-1 in blood leukocyte DNA and gastric cancer risk in a case–control study (Hou *et al*, 2010). To follow-up this lead, we conducted a nested case–control study within the Shanghai Women's Health Study (SWHS) to prospectively examine the relationship between gastric cancer risk and DNA methylation in LINE-1 and Alu regions from pre-diagnostic blood leukocytes.

Methylation reactions depend on the availability of methyl groups from *S*-adenosylmethionine, which are mainly derived from foods that contain methionine, folic acid, and choline (Lamprecht and Lipkin, 2003); intake deficiency of these foods has been associated with decreased methylation level in colon and gastric tissues (Kim *et al*, 2003; Galvan-Portillo *et al*, 2009). Other lifestyle factors, such as alcohol drinking, cigarette smoking, and vitamins use, have also been linked to DNA methylation change and related with cancer status (Nan *et al*, 2005; Hillemacher *et al*, 2008). Furthermore, carriage of *H. pylori* has been suggested to interact with DNA methylation and lead to gastric carcinogenesis (Chan *et al*, 2003, 2007; Ushiku *et al*, 2007; Yamamoto *et al*, 2008; Hino *et al*, 2009). We also explored effect modification by these *a priori* factors on the methylation–cancer association.

## Materials and methods

### Study population

The SWHS; Zheng *et al*, 2005) is a prospective cohort comprised of 74 942 women, aged 40–70 years at study entry (1997–2000), from urban Shanghai, China. Participants were interviewed (response rate 93%) to elicit information on demographic characteristics, height and weight history, medical history, family cancer history, menstrual and reproductive history, dietary habits, and other lifestyle factors. Dietary intake was ascertained by a validated food-frequency questionnaire that included frequency of consumption (daily, weekly, monthly, yearly, or not at all) and portion size of each serving (Epplein *et al*, 2010). Daily consumption of isoflavone and folic acid from food was calculated according to the nutrient content per gram of the food obtained from the Chinese Food Composition Tables (Lee *et al*, 2009; Epplein *et al*, 2010). In addition, 56 831 participants provided a 10-ml blood sample collected into an EDTA vacutainer tube. Blood samples were kept at 0–4°C, transported on ice, and saved at −70°C within 6 h after collection. DNA was extracted from blood leukocytes with the standard phenol–chloroform method. Incident cancer patients were identified by biennial in-person follow-up interview and by annual linkage to the population-based Shanghai Cancer Registry.

All incident gastric cancer cases (*n*=192) diagnosed through December 2009, who provided a blood sample, were included in the current project. For each case, two cancer-free controls were randomly selected from cohort members and matched to the cases by age (±2 years), menopausal status at sample collection, date (±30 days) and time (morning or afternoon) of blood draw, and time since last meal (±2 h).

This study was approved by the institutional review boards of all participating study centers in China and the United States. All participants provided written informed consent.

### DNA methylation measurement

For the current study, 500 ng DNA extracted from baseline buffy coat sample for each case and control was treated with bisulphite to convert unmethylated cytosine to uracil. Each assay was compared with an internal control sample to monitor the completion of bisulphite conversion. The bisulphite-treated DNA was then used for PCR amplification of Alu and LINE-1 elements, followed by pyrosequencing, a highly quantitative method as previously described (Tarantini *et al*, 2009; Baccarelli *et al*, 2010), to measure the degree of methylation for LINE-1 and Alu. Methylation status was expressed as the percentage of methylated cytosines over the total of methylated and unmethylated cytosines.

All of the 576 samples were tested in eight batches/plates. Matched case and control trio samples were placed within the same batch in random order. One negative control was included in each batch. To assess variability of the assay, duplicates from each of two QC subjects were randomly and blindly dispersed among study samples in each of eight batches (total 32 QC samples). Duplicate assays from bisulphate-converted DNA samples were analysed for each subject and the average was used for statistical analysis. The analytical variability (coefficient of variation calculated from replicates of the QC samples) was 2.2% and 1.0% for Alu methylation and LINE-1 methylation, respectively.

### Statistical analysis

DNA methylation levels were classified as quartiles for statistical analysis according to their distributions in controls, and the highest quartile was used as the reference group for risk estimation. Logistic regression was used to estimate odds ratios (ORs) and 95% confidence intervals (95% CI) for the association between DNA methylation and gastric cancer risk. As there was essentially no difference in the risk estimates between conditional and unconditional logistic regressions, we reported results from unconditional logistic regression. Adjustment for age, body mass index (BMI), education, family history of gastric cancer, menopause status, cigarette smoking, alcohol drinking, and use of antibiotics and cold medicine 1 week before blood draw did not modify the risk estimates appreciably (<5%). Among a subset of subjects (67% of total subjects) with *H. pylori* infection status, further adjustment for this variable did not alter risk estimates either. Therefore, the results were reported from logistic regression adjusted for age only.

Taking advantage of the prospective design of the SWHS, we explored the effect of latency on the association between DNA methylation and gastric cancer risk. For cases, latency was defined as the time difference between blood draw and gastric cancer diagnosis; for controls, latency was defined as the time difference from blood draw to the time their corresponding cases was diagnosed with gastric cancer.

To explore whether the methylation-cancer association was modified by selected exposure factors, we conducted analyses stratified by family history of gastric cancer, menopause status, tea drinking, dietary intake of isoflavone and folic acid, and intake of vegetable and fruit. Dietary intake was dichotomously classified into high *vs* low intake based on the median in controls. Interactions were evaluated with log-likelihood ratio test comparing models with and without the interaction term. All statistical analyses were conducted with SAS 9.1 statistical software (Rockville, MD, USA) and all tests were two-sided. An analysis with *P*-value <0.05 was considered statistically significant.

## Results

A total of 192 gastric cancer cases and 384 individually matched controls were included in the current analyses. Cases and controls were similar with regard to age, BMI, education, family history of gastric cancer, menopause status, recent non-steroidal anti-inflammatory drug and antibiotics use, vitamin B use, tea drinking, and dietary intake (isoflavones, folic acid, vegetables, and fruits; Table 1). Among subjects with information on *H. pylori* infection status, over 90% of cases and controls were positive. Smoking and alcohol drinking were not prevalent in Chinese women. The median levels of Alu and LINE-1 were nearly identical in cases and controls.

Compared with the highest quartile, decreased Alu methylation was associated with increased gastric cancer risk overall, with risk estimates of 2.17 (95% CI=1.32–3.56), 1.37 (95% CI=0.82–2.31), and 1.85 (95% CI=1.10–3.11) for the third, second, and the lowest quartiles, respectively. When we stratified the analysis by latency, however, we found that the associations for cases diagnosed within the first year following blood draw were different from those for cases diagnosed at a later time (Table 2). Within the first year of blood draw, hypomethylation of Alu appeared inversely associated with gastric cancer risk, although this observation was based on small numbers of subjects and none of the risk estimates were statistically significant (Table 2). For individuals whose gastric cancer was diagnosed at ⩾1 years after blood collection, risk was elevated with hypomethylation of Alu, although not in a consistent manner with either progression in hypomethylation or increasing years since blood draw. For LINE-1 hypomethylation, there were no consistent associations with gastric cancer risk, regardless of the years since blood collection.

We explored whether selected exposures might modify the association between hypomethylation in Alu and gastric cancer risk. For this analysis, we excluded cases diagnosed within 1 year of blood collection and their controls to minimise the potential impact of preclinical cancer. The inverse associations between the gastric cancer risk and Alu methylation tended to be stronger among women who consumed tea regularly and had high levels of intake of isoflavone, folic acid, or vegetable (Table 3). However, only the interaction with dietary isoflavone intake reached statistical significance (*P*_interaction_=0.04). In contrast, there were no consistent patterns of effect modification of the association between LINE-1 methylation and gastric cancer risk by selected exposures (Table 4).

## Discussion

In this exploratory study, we observed inverse associations between gastric cancer risk and methylation of Alu, but not LINE-1, in pre diagnostic blood leukocytes. The association with Alu methylation appeared to be modified by years since blood collection, with an inverse association observed only for cases diagnosed ⩾1 year after blood collection. Furthermore, this association appeared to be modified by dietary intake, particularly intake of isoflavone.

Our study has several advantages and limitations. First, the study is nested within the population-based SWHS cohort, which is a well-characterised population of women at high risk for gastric cancer. Second, DNA obtained from blood samples at baseline allowed us to assess methylation status before cancer diagnosis, and explore the latency effect on the associations. To our knowledge, this is among the first prospective population-based study to date to examine the association between DNA methylation and cancer risk. In addition, detailed exposure information (i.e., dietary and other life style factors) allowed us to explore potential effect modifications. However, the sample size is relatively small, and we had limited power to detect interactions with potential effect modifiers. Besides, if donated blood samples of the participants of SWHS (75.8% of all participants) were different from those who did not donate blood, it could limit the generalisability of our results to those who did not provide a blood sample.

To date, a few case–control studies have examined the association between global DNA methylation in leukocytes and risk of various cancers, including cancers of the head and neck, stomach, breast, colon and rectum, and bladder (Pufulete *et al*, 2003; Hsiung *et al*, 2007; Moore *et al*, 2008; Choi *et al*, 2009; Hou *et al*, 2010; Wilhelm *et al*, 2010; Cash *et al*, 2011). Though all of the studies suggested an inverse association between global DNA methylation and cancer risk, not all results were statistically significant (Pufulete *et al*, 2003; Choi *et al*, 2009; Cash *et al*, 2011). Only one study examined gastric cancer risk in relation to both Alu and LINE-1 methylation in a Polish case–control study and found insignificant inverse associations with both (Hou *et al*, 2010). We did not observe a consistent dose-response effect with Alu methylation, raising the possibility of chance variations. However, the lack of linear association with methylation status has been reported previously, such as the association between bladder cancer and global DNA methylation evaluated directly with 5′-methylcytosine (Moore *et al*, 2008), which might reflect a threshold effect. Further evidence is needed to clarify the association between peripheral leukocyte DNA methylation and gastric cancer risk.

Though not statistically significant, we found that the association between Alu methylation and gastric cancer risk was stronger among individuals with longer latency. Certainly, this exploratory finding warrants further research. Ideally, it would be most informative if blood samples were available to monitor changes in DNA methylation for the same persons at multiple time points before cancer diagnosis.

We explored effect modification by several *a priori* factors and found a significant interaction only with dietary isoflavone intake, with a stronger association between gastric cancer risk and Alu hypomethylation among individuals with high isoflavone intake. This observation appears inconsistent with the known inhibitory effect of isoflavones on cancer development (Gilbert and Liu, 2010). It is also possible that this apparent effect modification is a chance occurrence.

Retro transposition (mobile genetic elements inserting into a new location) of LINE-1 and Alu has been reported to increase genomic instability, activate endogenous parasitic sequence, and contribute to the transcriptional silencing of tumour-suppressor genes in tissues, thus, leading to tumour development (Esteller and Herman, 2002; Gilbert *et al*, 2002; Symer *et al*, 2002). In addition, Alu and LINE-1 may also participate in regulating immune and inflammatory responses (Li and Schmid, 2001; Hagan and Rudin, 2002). Emerging data from epidemiological studies suggest different functional roles of Alu and LINE-1 in cancer initiation and progression (Bollati *et al*, 2007; Rusiecki *et al*, 2008; Jintaridth and Mutirangura, 2010). Though Alu and LINE-1 were correlated to each other in tumour tissue (Choi *et al*, 2007), a correlation has not always been observed inblood (Bollati *et al*, 2007; Rusiecki *et al*, 2008). A study on breast cancer found increased cancer risk associated with lower 5-mdC level in leukocyte DNA but not LINE-1 methylation level; further, LINE-1 methylation was not correlated with 5-mdC (Choi *et al*, 2009). Direct measurement of global 5-mdC is still needed to examine the role of global DNA methylation in gastric cancer aetiology. In the present study, we observed significantly increased risk of gastric cancer associated with hypomethylation of Alu but not LINE-1, and the correlation between LINE-1 methylation and Alu methylation among controls was modest (correlation coefficient of determination *R*^2^=0.12). Our findings seem to support the notion that Alu and LINE-1have different roles in gastric carcinogenesis, but given the small numbers in this study, we cannot rule out chance as an explanation.

In summary, the results from this prospective study suggest that DNA methylation of Alu, but not LINE-1, in blood leukocytes might be inversely associated with risk of gastric cancer. The divergent associations with Alu and LINE-1 raise the possibility that the roles of DNA methylation in these two elements are different in gastric cancer aetiology. However, replication is needed before further inference may be drawn from these observations.

## Figures and Tables

**Table 1:** Characteristics at baseline for gastric cancer cases and controls nested in the Shanghai Women's Health Study

**Characteristics^a^**	**Controls (*n*=384)**	**Cases (*n*=192)**	***P* b**
Age (year)	61 (51–65)	61 (50–65)	0.97
			
*Education*			
College/higher	39 (10.2)	12 (6.2)	0.27
High school	82 (21.4)	35 (18.2)	
Middle school	109 (28.4)	57 (32.7)	
Elementary/less	154 (40.1)	88 (45.8)	
			
Family history of gastric cancer	28 (7.3)	16 (8.3)	0.66
Menopause	311 (81.0)	159 (83.2)	0.51
*H. pylori* positivity^c^	260 (92.2)	136 (96.4)	0.09
Ever smoking	19 (5.0)	12 (6.2)	0.52
Ever alcohol use	8 (2.1)	3 (1.6)	0.67
NSAID/cold medication use within past week	36 (9.4)	12 (6.2)	0.20
Antibiotics use within past week	31 (8.1)	9 (4.7)	0.13
Vitamin B supplement use within past year	21 (5.5)	10 (5.2)	0.89
Tea drink regularly	111 (28.9)	43 (22.4)	0.10
			
Body mass index (kg m^−2^)	24.3 ((22.4–26.5)	24.6 (22.3–27.3)	0.53
Dietary isoflavones intake (ug per day)	37.6 (21.7–57.7)	39.0 (20.9–53.3)	0.90
Dietary folic acid intake (ug per day)	280.7 (218.9–346.0)	286.3 (211.4–348.2)	0.73
Vegetables intake (g per day)	262.0 (178.7–366.3)	242.1 (155.5–371.0)	0.18
Fruit intake (g per day)	221.3 (121.8–352.3)	207.5 (95.9–323.9)	0.16
			
Alu methylation (%)	25.3 (25.0–25.6)	25.3 (24.95–25.5)	
LINE-1 methylation (%)	85.2 (84.6–85.7)	85.1 (84.6–85.8)	

Abbreviations: *H. pylori=Helicobacter pylori*; LINE=long interspersed nucleotide elements; NSAID=non-steroidal anti-inflammatory drug.

a Continuous variables are displayed as median (interquartile) and categorical variables are displayed as frequency (percentage among cases or controls).

b *P*-values are calculated from paired *t*-test for continuous variable and *χ*^2^-test for categorical variables.

c *H. pylori* status was only available for 423 subjects (141 cases and 282 controls).

**Table 2:** Association of Alu and LINE-1 methylation levels in blood leukocytes and risk of gastric cancer by latency^a^

	**Q4**		**Q3**		**Q2**		**Q1**	
	***N*_case/control_**	**95% CI**	***N*_case/control_**	**95% CI**	***N*_case/control_**	**95% CI**	***N*_case/control_**	**95% CI**
Alu (%)	⩾25.6		25.3–25.6		25.0–25.2		<25.0	
Overall	36/111	1	64/91	**2.17** (**1.32–3.56)**	44/99	1.37 (0.82–2.31)	48/80	**1.85** (**1.10–3.11)**
<1 years	6/9	1	6/10	0.83 (0.17–4.15)	5/9	0.78 (0.16–3.91)	1/8	0.18 (0.02–1.91)
1–4.99 years	15/48	1	23/36	2.04 (0.94–4.46)	17/35	1.56 (0.69–3.54)	19/30	2.04 (0.90–4.62)
⩾5years	15/54	1	35/45	2.80 (1.36–5.78)	22/55	1.44 (0.68–3.07)	28/42	**2.40** (**1.14–5.07)**
*P*_interaction_^b^								0.30
								
LINE-1 (%)	⩾85.7		85.1–85.6		84.5–85.0		<84.5	
Overall	57/104	1	36/90	0.73 (0.44–1.21)	58/104	1.02 (0.64–1.60)	41/84	0.89 (0.54–1.46)
<1 years	3/7	1	7/12	1.36 (0.26–7.05)	6/10	1.40 (0.26–7.59)	2/6	0.78 (0.10–6.32)
1–4.99 years	14/13	1	13/35	0.60 (0.26–1.37)	18/40	0.71 (0.33–1.52)	19/38	079 (0.36–1.70)
⩾5years	30/59	1	16/44	0.71 (0.35–1.47)	34/54	1.23 (0.67–2.29)	20/40	0.98 (0.49–1.97)
*P*_interaction_^b^								0.80

Abbreviations: CI=confidence interval; LINE=long interspersed nucleotide elements.

a Adjusted for age; latency was defined as the time from blood draw to cancer diagnosis for cases, and the same value was assigned to the matched controls.

b *P*-value for interaction tested by coding the Alu and LINE-1 quartiles as ordinal variable (1,2,3,4). Bold entries indicate *P*<0.05.

**Table 3:** Association of Alu methylation levels in blood leukocytes and risk of gastric cancer by potential effect modifiers^a^

	**Q4**		**Q3**		**Q2**		**Q1**		
	***N*_case/control_**	**OR (95%)**	***N*_case/control_**	**OR (95%)**	***N*_case/control_**	**OR (95%)**	***N*_case/control_**	**OR (95%)**	***P* ^b^**
Alu	30/102	1	58/81	**2.43** (**1.43–4.13)**	39/90	1.47 (0.85–2.57)	47/72	**2.22** (**1.28–3.84)**	
Family history (yes)	3/9	1	4/5	2.71 (0.41–18.2)	3/5	1.99 (0.28–14.3)	5/7	2.42 (0.40–14.4)	0.92
Family history (no)	27/93	1	54/76	**2.45** (**1.41–4.25)**	36/85	1.46 (0.82–2.61)	42/65	**2.23** (**1.25–3.97)**	
									
Menopause (yes)	22/82	1	49/62	**2.93** (**1.60–5.34)**	37/77	1.78 (0.96–3.29)	38/62	**2.27** (**1.22–4.23)**	0.99
Menopause (no)	8/20	1	9/19	1.31 (0.41–4.20)	2/13	0.35 (0.06–1.95)	9/10	2.18 (0.64–7.42)	
									
Tea (yes)	2/28	1	17/28	**8.54** (**1.80–40.5)**	13/24	**7.59** (**1.56–37.1)**	10/21	**6.73** (**1.33–34.1)**	0.31
Tea (no)	28/74	1	41/53	**2.04** (**1.13–3.71)**	26/66	1.04 (0.56–1.95)	37/51	1.92 (1.04–3.52)	
									
Isoflavone (high)	20/45	1	27/38	**4.11** (**1.82–9.29)**	18/46	**2.72** (**1.16–6.35)**	20/37	**4.37** (**1.89–10.1)**	**0.04**
Isflavone (low)	10/57	1	31/43	1.59 (0.77–3.27)	21/44	0.88 (0.42–1.89)	27/35	1.21 (0.57–2.58)	
									
Folic acid (high)	18/47	1	25/43	**3.98** (**1.82–8.68)**	18/42	1.98 (0.88–4.45)	21/38	**3.47** (**1.54–7.78)**	0.26
Folic acid (low)	12/55	1	33/38	1.50 (0.72–3.13)	21/48	1.11 (0.51–2.41)	26/34	1.42 (0.66–3.05)	
									
Vegetable (high)	17/50	1	36/46	**2.54** (**1.13–5.70)**	19/42	1.66 (0.74–3.70)	26/39	2.53 (1.12–5.74)	0.56
Vegetable (low)	13/52	1	22/35	**2.30** (**1.14–4.64)**	20/48	1.34 (0.62–2.90)	21/33	1.95 (0.93–4.10)	
									
Fruit (high)	12/44	1	31/41	**2.17** (**1.06–4.46)**	18/47	1.58 (0.75–3.33)	24/43	**2.58** (**1.20–5.53)**	0.41
Fruit (low)	18/58	1	27/40	**2.80** (**1.26–6.17)**	21/43	1.41 (0.61–3.25)	23/29	2.07 (0.92–4.68)	

Abbreviation: OR=odds ratio.

a Adjusted for age ; subjects with latency less than 1 year were excluded.

b *P*-value for interaction tested by coding the Alu as ordinal variable (1,2,3,4). Bold entries indicate *P*<0.05.

**Table 4:** Association of LINE-1 methylation levels in blood leukocytes and risk of gastric cancer by potential effect modifiers^a^

	**Q4**		**Q3**		**Q2**		**Q1**		
	***N*_case/control_**	**OR (95%)**	***N*_case/control_**	**OR (95%)**	***N*_case/control_**	**OR (95%)**	***N*_case/control_**	**OR (95%)**	***P*** b
LINE-1	54/97	1	29/78	0.67 (0.39–1.15)	52/94	0.99 (0.62–1.60)	39/78	0.90 (0.54–1.50)	
Family history(yes)	4/5	1	2/12	0.13 (0.01–1.55)	4/5	0.55 (0.09–3.57)	5/4	1.74 (0.27–11.1)	0.26
Family history (no)	50/92	1	38/82	0.75 (0.43–1.30)	45/83	1.03 (0.63–1.68)	26/64	0.82 (0.48–1.41)	
									
Menopause (yes)	44/78	1	36/77	0.70 (0.39–1.26)	41/73	1.03 (0.61–1.74)	25/55	0.90 (0.51–1.58)	0.98
Menopause (no)	10/19	1	4/17	0.46 (0.11–2.04)	8/15	0.83 (0.26–2.59)	6/13	0.96 (0.29–3.15)	
									
Tea (yes)	16/29	1	6/22	0.35 (0.10–1.18)	13/27	1.08 (0.43–2.70)	7/25	0.52 (0.20–1.37)	0.34
Tea (no)	38/68	1	34/72	0.78 (0.42–1.45)	36/61	0.97 (0.56–1.70)	24/43	1.14 (0.62–2.10)	
									
Isoflavone (high)	25/44	1	18/50	0.84 (0.40–1.77)	30/42	0.85 (0.44–1.66)	12/31	0.88 (0.44–1.78)	0.65
Isoflavone (low)	29/53	1	22/44	0.54 (0.24–1.18)	19/46	1.15 (0.58–2.27)	19/37	0.91 (0.43–1.91)	
									
Folic acid (high)	24/52	1	16/43	0.61 (0.30–1.26)	29/43	0.75 (0.38–1.48)	13/33	0.74 (0.36–1.50)	0.36
Folic acid (low)	30/45	1	24/51	0.70 (0.31–1.62)	20/45	1.28 (0.65–2.52)	18/35	1.08 (0.52–2.26)	
									
Vegetable (high)	28/53	1	22/40	0.41 (0.18–0.92)	31/46	0.91 (0.46–1.82)	17/39	0.71 (0.32–1.58)	0.74
Vegetable (low)	26/44	1	18/54	1.08 (0.52–2.26)	18/42	1.06 (0.55–2.04)	14/29	1.06 (0.54–2.08)	
									
Fruit (high)	21/47	1	18/44	0.56 (0.27–1.16)	27/50	0.90 (0.46–1.76)	19/33	0.60 (0.28–1.26)	0.16
Fruit (low)	33/50	1	22/50	0.83 (0.37–1.89)	22/38	1.16 (0.58–2.33)	12/35	1.34 (0.65–2.76)	

Abbreviations: OR=odds ratio; LINE=long interspersed nucleotide elements.

a Adjusted for age; subjects with latency <1 year were excluded.

b *P*-value for interaction tested by coding the LINE-1 as ordinal variable (1,2,3,4).
